# Antioxidant, Anti-Inflammatory Effects and Ability to Stimulate Wound Healing of a Common-Plantain Extract in Alginate Gel Formulations

**DOI:** 10.3390/gels9110901

**Published:** 2023-11-14

**Authors:** Ioana Bâldea, Ildiko Lung, Ocsana Opriş, Adina Stegarescu, Irina Kacso, Maria-Loredana Soran

**Affiliations:** 1 Department of Physiology, Iuliu Haţieganu University of Medicine and Pharmacy, Clinicilor 1, 400006 Cluj-Napoca, Romania; baldeaioana@gmail.com; 2 National Institute for Research and Development of Isotopic and Molecular Technologies, 67-103 Donat, 400293 Cluj-Napoca, Romania; ildiko.lung@itim-cj.ro (I.L.); ocsana.opris@itim-cj.ro (O.O.); adina.stegarescu@itim-cj.ro (A.S.); irina.kacso@itim-cj.ro (I.K.)

**Keywords:** alginate beads, anti-inflammatory effect, antioxidant capacity, plantain, polyphenols, wound healing

## Abstract

Our study aimed to investigate the biological effects of a common-plantain (*Plantago major* L.) extract, encapsulated in alginate, on dermal human fibroblast cultures *in vitro*, in view of its potential use as a wound healing adjuvant therapy. Common-plantain extracts were obtained by infusion and ultrasound extraction, and their total polyphenolic content and antioxidant capacity were determined by spectrophotometry. The best extract, which was obtained by infusion, was further encapsulated in sodium alginate in two different formulations. Fourier Transform Infrared Spectroscopy (FTIR) was used to demonstrate the existing interactions in the obtained common-plantain extract in the alginate formulations. The encapsulation efficiency was evaluated based on the total polyphenol content. These alginate gel formulations were further used *in vitro* to determine their biocompatibility and antioxidant and anti-inflammatory effects by spectrophotometry and ELISA, as well as their ability to stimulate fibroblast migration (scratch test assay) at different time points. In addition, the collagen 1 and 3 levels were determined by Western blot analysis. The data showed that the microencapsulated plantain extract formulations induced an antioxidant, anti-inflammatory effect, enhanced collagen production and increased wound closure in the first 8 h of their application. These results are encouraging for the use of this alginate plantain extract formulation as an adjuvant for skin wound healing.

## 1. Introduction

The study of plant-derived biologically active compounds is still a rather unexploited area, despite the accumulating data showing that medicinal plants contain an array of active molecules, such as vitamins, flavonoids, terpenes, phytoncides and glycosides. These compounds have several therapeutic properties such as pulmonary protective potential, a neuroprotective effect, etc. [1,2,3,4,5,6]. A big issue is that clinical studies of wound-healing mechanisms triggered by nonchemical substances are not acceptable due to ethical reasons. The main question that remains is which compounds extracted from plants are the most efficient and safe for use in the treatment of certain clinical types of skin wounds, especially, chronic or nonhealing wounds. For those reasons, in the last years, the research community tried to develop new different assays *in vitro*, *in vivo* and *ex vivo* to allow a preclinical screening of such compounds for biocompatibility and efficacy in wound healing [7,8]. *Plantago major*, commonly known as plantain, has been used in traditional treatments for wound healing, but the mechanisms underlining its beneficial effects are not known [9]. There are few studies that investigated the potential of plantain-containing formulations for wound healing. 

Krasnov et al., in 2011, showed that proteins isolated from plantain acted as bioregulators, increased wound healing in an experimental model of skin tissue *in vitro* and induced skin wound healing effects *in vitro* and *in vivo* [10].

Zubairab et al., in 2015, reported a Plantago major-based product that could stimulate wound healing using a porcine wound model. The study showed that ethanol- and water-based extracts stimulated wound healing in the *ex vivo* pig skin following exposure to three concentrations, i.e., 0.01, 0.1 and 1.0 mg mL^−1^ [8].

Plant extracts are not very stable under any conditions; therefore, they were encapsulated in hydrogels and further tested for their effects in skin regeneration [11,12,13]. Hydrogels represent a class of three-dimensional polymeric networks with a macroporous structure that allows proper cellular interactions with the host tissue. They have a specific porosity that allows the diffusion and transport of external nutrients to the target [11,12,13]. 

Due to the properties of hydrogels, like hydrogen bonding between the polymer chains, weak connections (covalent bonding) within the polymer networks, hydrogels resist to permanent deformation and retain a high content of water. All this results in a delicate softness, allowing hydrogels to mimic natural tissues [14,15,16,17].

Masood et al., in 2022, reported for the first time the synthesis of hydrogels consisting of chitosan and chondroitin sulfate combined with a garlic extract. They investigated a novel combination of chitosan and chondroitin sulphate for synthesizing hydrogels using a freeze gelation process and, after that, enriched the obtained hydrogel with a garlic extract. This complex proved to be biocompatible, increased the speed of wound healing and provided resistance against *Escherichia coli* and *Staphylococcus aureus* growth on the surface of the wound. These hydrogels with/without garlic loading showed high water retention and good dissolution ratio when in contact with biological tissues, which are useful properties for local drug delivery [18]. In addition, chitosan-based hydrogels were investigated by Zahid et al. in 2021. They obtained and characterized chitosan/*Calotropis procera* (latex extract)-based hydrogels which stimulated the appearance of granulation tissue and increased angiogenesis, which are crucial stages of wound healing [19]. 

In another interesting study recently published by Kȩdzierska et al., research was focused on obtaining and characterizing new hydrogel dressings containing both a licorice root extract and nano silver, a combination that prevents inflammation and infection within wounds. The results confirmed the stability of the tested hydrogels in synthetic physiological liquids. A great sorption ability was observed that indicated the possibility of absorbing a wound exudate. Surface hydrophilicity and elasticity (approximately, 30% flexibility) indicated the possible use of these developed hydrogels as dressing materials to support regenerative processes in tissue injury [20,21].

*Aloe vera* mucilaginous-based hydrogels showed a great potential for the topical treatment of psoriasis plaques due to their keratolytic action, but more studies are required to ensure their safety in clinical practice [22]. 

In traditional Chinese medicine, curcuminoids are used for the treatment of burns and other wounds. Microencapsulation of curcuminoids using gelatin B and chitosan showed a significant effect on wound healing, with increased wound closure rate and better epithelialization [23].

In a recent report [24], an herbal hydrogel formulation of *Moringa oleifera* obtained from a hexane extract of *M. oleifera* seeds showed antibacterial activity against both Gram-positive and Gram-negative species combined with wound closure promotion in both excision and incision models in mice, due to the induction of mitogenesis by the hexane hydrogel of *M. oleifera* seeds [24]. 

More studies are needed in the future to allow the implementation of these plant extracts encapsulated in hydrogels as efficient alternative solutions in the treatment of wound healing and skin diseases.

Here, we report for the first time the preparation of hydrogels using *Plantago major* extracts. This study aimed to evaluate *in vitro* on human dermal fibroblasts the biocompatibility and potential to promote skin wound healing of a common-plantain extract encapsulated in a sodium alginate hydrogel, by the assessment of the wound area at different time points using a wound scratch assay and the evaluation of the hydrogel’s antioxidant and anti-inflammatory effects as well as of the synthesis of collagen, an essential extracellular matrix protein supporting the skin repair process. Additionally, sodium alginate beads and plantain extracts are green products that were obtained by ecofriendly methods, which further sustains the validity of the described approach. The obtained hydrogel with the encapsulated plantain extract is intended as an adjuvant therapy for chronic or nonhealing wounds. 

## 2. Results and Discussion

### 2.1. Extract Analysis

**Figure 1:**
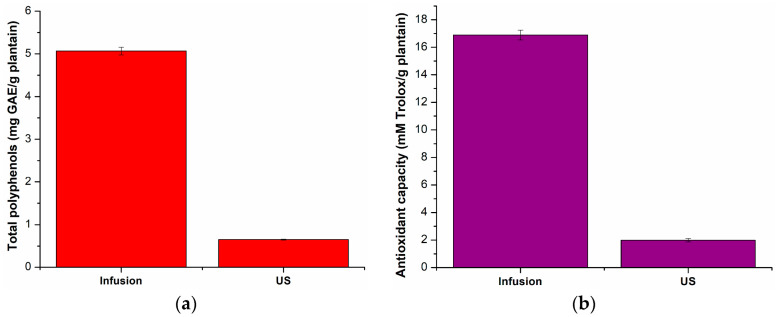
Total polyphenol content (**a**) and antioxidant activity (**b**). Each data point is the mean ± the standard error of the mean of three independent experiments.

The total content of polyphenolic compounds in extracts of common-plantain dried leaves was determined using the equation of the gallic acid equivalent (GAE) calibration curve, i.e., y = 0.577x + 0.0073 (R^2^ = 0.9992), and the results are presented in Figure 1a.

As presented in Figure 1a, the highest amount of polyphenols was obtained when using infusion as the extraction technique. With this method, 5.07 mg GAE/g of plantain was extracted, compared to 0.65 mg GAE/g of plantain obtained with ultrasound extraction.

Mazzutti et al. [25] found in their extracts a total amount of polyphenols that varied between 2 and 121 mg GAE/g of extract. This variation depended on the type of method used for the extract preparation (supercritical fluid extraction, Soxhlet extraction or ultrasound extraction), as well as on the extraction solvent. The smallest amount of polyphenols was obtained with supercritical fluid extraction (CO_2_, 10 MPa, 40 °C), and the largest amount of polyphenols was obtained with extraction by sonication, using as the extraction solvent a mixture of ethanol and water (70:30, *v*/*v*).

Subcritical water extraction and microwave-assisted water extraction were investigated for total polyphenol extraction from common plantain [26]. Both types of extraction were carried out at several temperatures, ranging from 25 to 200 °C in the case of subcritical water extraction and from 50 to 200 °C in the case of microwave-assisted water extraction. The total polyphenolic amount ranged from 35.3 to 113.2 mg GAE/g of extract for subcritical water extraction and from 41.9 to 102.6 mg GAE/g of extract for microwave-assisted water extraction. In both cases, the total amount of polyphenols increased with the temperature. 

The total polyphenolic content of the extracts obtained by maceration was determined according to the Folin–Ciocalteu procedure. The total polyphenolic content was 5.79 mg GAE/g of extract for a petroleum ether extract, 26.89 mg GAE/g of extract for an aqueous extract and 114.45 mg GAE/g of extract for an ethyl acetate extract [27]. 

The antioxidant capacity of the common-plantain extracts was determined from the Trolox calibration curve y = 0.1831x + 0.0091 (R^2^ = 0.9993), and the results are presented in Figure 1b. According to this calculation, the highest antioxidant capacity of the extracts (16.90 mM Trolox/g of plantain) was obtained when infusion was performed. 

Considering the total amount of polyphenols and the antioxidant capacity of the common-plantain extracts obtained by the two methods used in this study, the extract obtained by infusion was chosen for encapsulation in sodium alginate.

### 2.2. Microencapsulated PE Characterization

**Figure 2:**
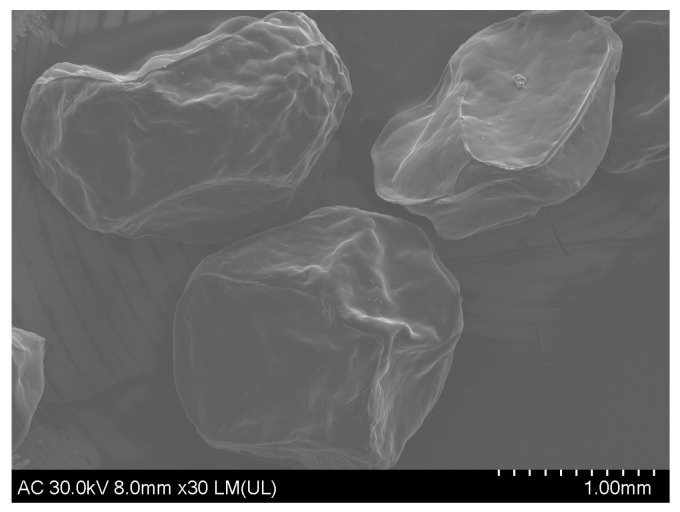
SEM micrographs of the microencapsulated PE sample.

The surface of the beads, as shown by SEM (Figure 2), was smooth, with small protuberances and holes that allowed the controlled release of the extract. The relative size of the beads was 1.8 ± 0.14/1.4 ± 0.1 mm.

**Figure 3:**
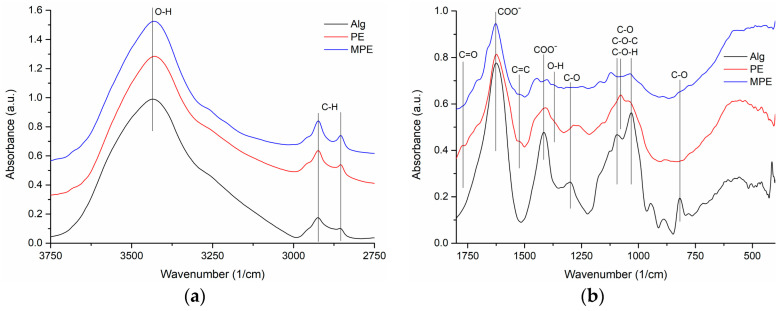
The FTIR spectra of Na alginate, common-plantain extract and microencapsulated common-plantain extract: (**a**) 3750–2750 cm^−1^; (**b**) 1800–400 cm^−1^ spectral domain.

The FT-IR spectra of the analyzed samples are presented in Figure 3.

By assigning the characteristic absorption peaks of Na alginate, the following vibrations were identified: at 3434 cm^−1^, the stretching vibrations of –OH groups, at 2924 and 2855 cm^−1^, the asymmetric and symmetric stretching of CH_2_ groups, at 1624 and 1416 cm^−1^, the asymmetric and symmetric stretching of –COO^-^ salt groups, at 1301 cm^−1^, the C–O stretching vibrations, at 1173 sh and 1124 cm^−1^, the stretching vibrations of C–C bonds, at 1095 and 1031 cm^−1^, the stretching vibrations of C–O and C–O–C groups in mannuronic and guluronic units [28], respectively, at 946 cm^−1^, the stretching of C–O groups from the pyranosyl ring, with contributions of C–C–H and C–O–H deformations, at 818 cm^−1^, the vibration of C–O groups in α-configuration in glucuronic units [29].

The FTIR spectrum of PE showed the following characteristic vibrational bands: at 3430 cm^−1^, the vibration of phenolic O–H, at 2923 and 2851 cm^−1^, the asymmetric and symmetric stretching of CH_2_ groups, at 1732sh cm^−1^, the stretching vibration of the carbonyl ––C=O group, at 1624 cm^−1^, the stretching vibration of the –COO^−^ group, at 1520sh cm^−1^, the stretching of aromatic –C=C–, at 1372sh cm^−1^, the bending vibration of O–H, at 1265 cm^−1^, the stretching of C–N and etheric C–O–C. The bands at 1158, 1078 and 1043sh cm^−1^ were assigned to the asymmetrical stretching of the C–C, C–O, C–O–C and C–O–H groups, and those at 990 and 774 cm^−1^ indicated C–H bending [30]. 

The MPE FTIR spectrum showed wider absorption bands with lower intensity, and the characteristic vibrational bands of the two components appeared slightly shifted. Thus, the –C=O stretching was shifted from 1732 cm^−1^ to 1744 cm^−1^, and the vibrations of the –C=C– group of the extract and of the –COO^−^ group of alginate were shifted from 1624 cm^−1^ to 1629 cm^−1^. The vibrational bands of PE at 1408 cm^−1^ with a shoulder at 1456 cm^−1^ and the band of alginate at 1416 cm^−1^ appeared as doublets, with peaks at 1447 and 1404 cm^−1^. The bands of PE at 1265 cm^−1^ and of alginate at 1300 cm^−1^ had a low intensity and were shifted at 1249 and 1072 cm^−1^, respectively, and a broad band with a maximum at 1086 cm^−1^ appeared. The bands of PE from 1079 to 1043 cm^−1^ and of alginate from 1094 to 1043 cm^−1^ were shifted on the spectrum of the mixture, appearing at 1122 and 1037 cm^−1^, respectively. The C–O vibration of alginate at 825 cm^−1^ presented a shoulder at 825 cm^−1^. All these changes suggested the existence of physical interactions between the components.

The encapsulation efficiency of MPE was 29.17 ± 0.03%. A low efficiency of encapsulation in sodium alginate was obtained for summer savory (4.79%) and rosemary (14.76%) extracts [31]. An encapsulation efficiency of 21% was obtained for an olive leave phenolic extract encapsulated in Alg beads [32].

### 2.3. Cell Viability

**Figure 4:**
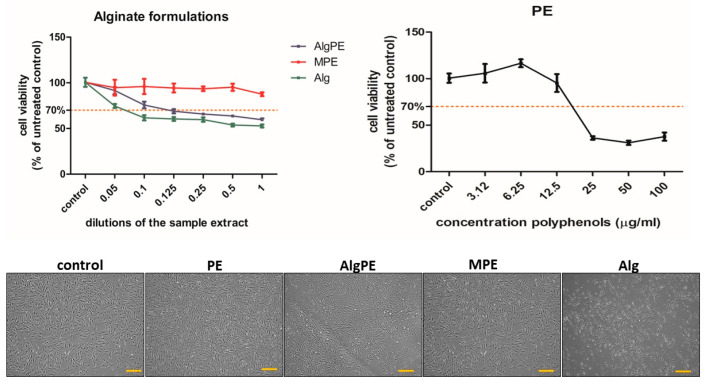
Cell viability of fibroblasts treated with alginate and PE formulations after 48 h of exposure to dilutions of each sample extract (**left panel**) and the PE extract with different concentrations of polyphenols (µg mL^−1^, **right panel**); the results are expressed as % of the viability of untreated controls, with the toxicity limit at 70%. Lower panels show representative microscopical images of the living cells in culture after 24 h exposure to different alginate formulations (dilution 1:20) and the PE extract (5 µg mL^−1^ of polyphenols), bar = 10 µm.

The PE extract (Figure 4) induced a decrease in fibroblast viability at concentrations of polyphenols above 12.5 µg mL^−1^. The alginate formulations induced different effects. MPE was very well tolerated at all concentrations used. AlgPE induced a dose-dependent decrease in cell viability. At concentrations above 0.1 of the sample extract (1:10), cell viability was below the toxicity level of 70% of that of the untreated control. Alginate, used as the vehicle control, decreased the viability of fibroblasts at all dilutions. Toxic effects were seen at concentrations above 0.05 (1:20). A decrease in fibroblast viability when applying high alginate concentrations was previously reported [33]. Also, very small concentrations of alginate of 2–5% were reported to be well tolerated by fibroblasts and allowed their proliferation [34]. Alginate was reported as a vehicle to immobilize cells, promote the secretion of extracellular matrix proteins by fibroblasts [35] and facilitate healing due to the prevention of fibroblast overgrowth in fibroblast–keratinocyte co-cultures. Moreover, alginate can be further used as a starting material for bioink formulations in 3D bioprinting and as a scaffold for tissue engineering [36,37]. The MPE formulation exhibited a very good biocompatibility, probably because of the low surface area of the spheres exposed to the medium, which diminished the amount of alginate that was extracted and interacted with the cells. The cell images taken after 48 h of exposure to a concentration of 5 µg mL^−1^ of polyphenols and different alginate formulations (1:20 dilution) showed that the morphology of the fibroblasts in culture was normal, with adherent, spindle-shaped cells, and cell confluence was lower in the alginate-treated samples.

### 2.4. Collagen Synthesis

**Figure 5:**
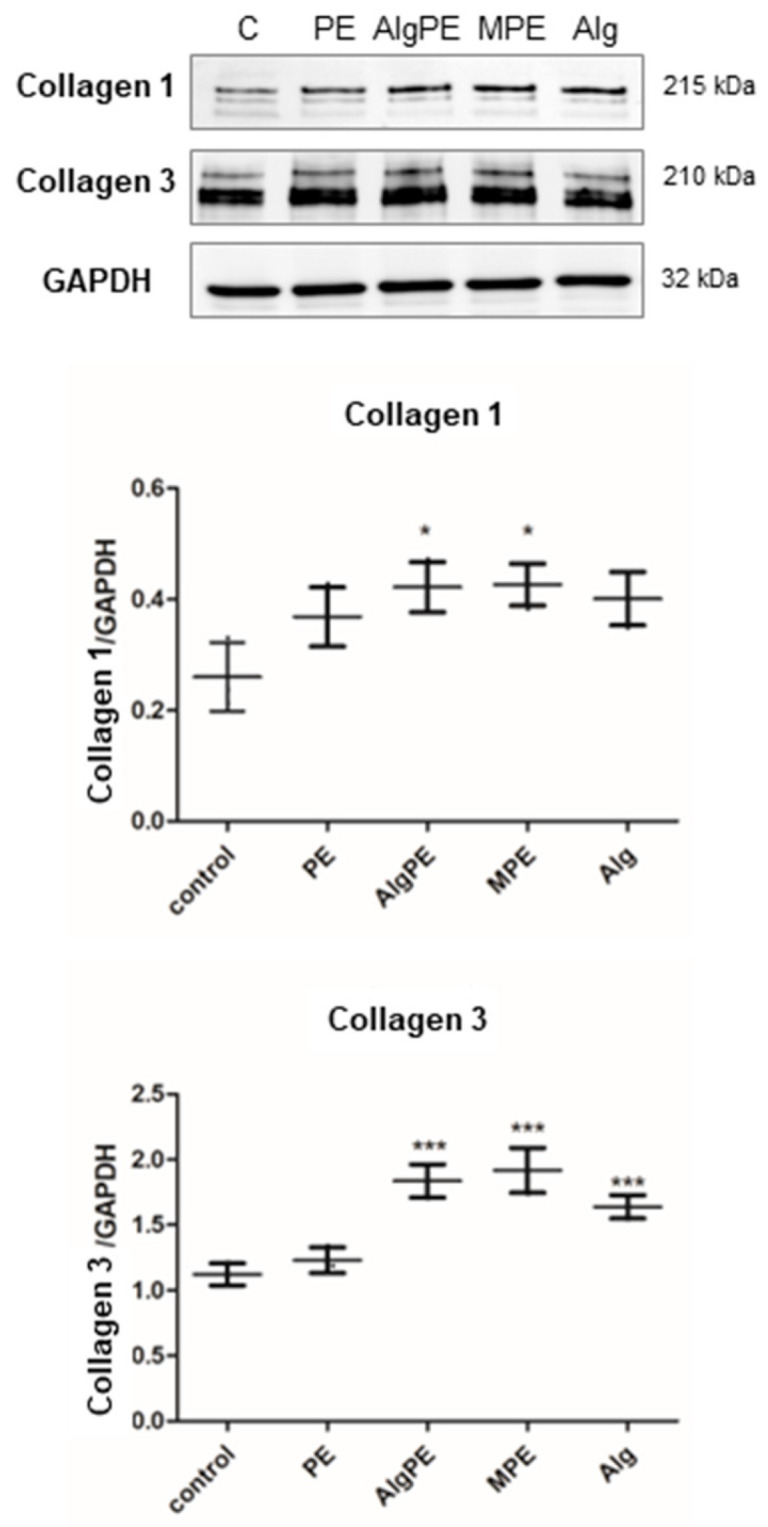
Collagen synthesis. Collagen 1 and 3 levels were determined by Western blot. Images (upper panel) were quantified by densitometry (lower panels), using GAPDH as a reference. Data are presented as the mean ± SD (*n* = 3); * = *p* < 0.05, *** *p* < 0.0001 compared to the control and between the treated groups.

Collagen, secreted by fibroblasts, is the most important molecule of the extracellular matrix and has a crucial role in wound healing. The synthesis of collagen (Figure 5) was investigated by WB, measuring the collagen content in the cell lysates. Collagen 1 synthesis was significantly increased by the exposure of the fibroblasts to AlgPE and MPE but not by the exposure to alginate and the PE extract. Overall, the Kruskal–Wallis test showed no significant differences between the groups (*p* = 0.089). The increase in ECM molecules in fibroblasts by alginate exposure was previously reported [34] and was found to be enhanced in the first 7 days of exposure; then the secretion diminished. Interestingly, the alginate PE formulations were more efficient in inducing collagen synthesis compared to alginate of the PE extract alone, which suggested an addictive effect in the two formulations. Collagen 3 synthesis was stimulated in all groups compared to the control, and the stimulation by all alginate formulations was significant. The most powerful effect was induced by MPE, and there was a significant statistical difference between MPE and alginate PE groups (*p* < 0.001). The Kruskal–Wallis test showed significant differences (*p* = 0.016). The results showed the ability alginate to sustain the viability of cells in culture, as well as to stimulate the formation of extracellular matrix proteins necessary for wound healing and for obtaining 3D cultures *in vitro*. Alginate formulations were previously investigated as potential bioinks [37,38] for the design of different scaffolds necessary for the 3D printing of bones [39], nerve tissues [40] and organoids [41], as well as for their antitumoral, antibacterial and antiviral properties [42].

### 2.5. Scratch Wound Assay 

**Figure 6:**
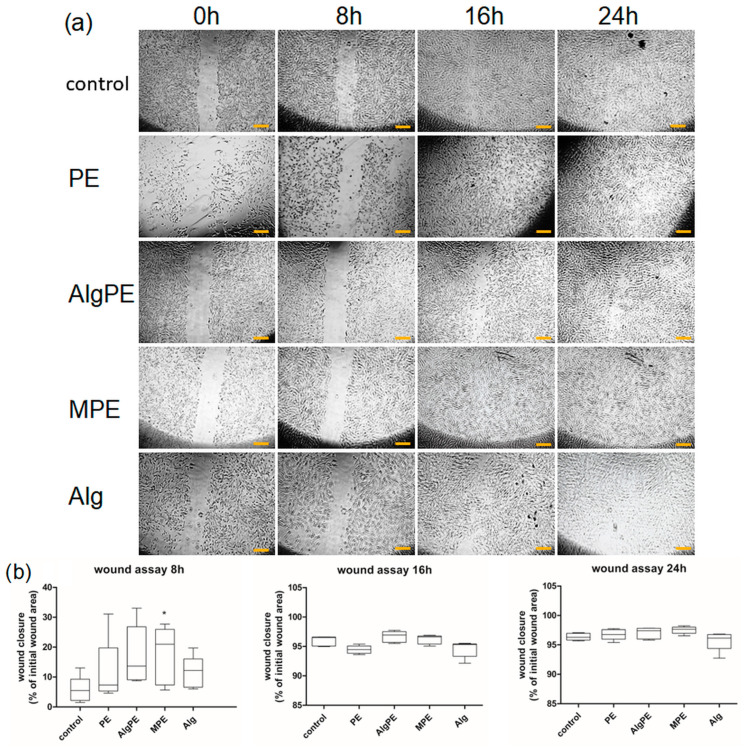
Scratch wound assay. (**a**) Comparative microscopic images of dermal fibroblasts (objective 4×) following exposure to the alginate formulations containing the extract (dilution 1:20) and to PE (5 µg mL^−1^ of polyphenols) at different time points, bar =10 µm; (**b**) quantification of the wound area closure was performed using the Image J software 1.8.0 and MiToBo plugging (2023). The results are presented as % of the initial wound area. The data are presented as mean ± SD (*n* = 3). * = *p* < 0.05 vs. control.

The migration of dermal fibroblasts was observed after the creation of a scratch wound (Figure 6a) for 30 h, until the wounds were fully closed in all groups. Cell images were taken initially and at 8 h, 16 h and 24 h. The quantification of cell migration showed different speeds of cell migration depending on the time point of the analysis and the treatment (Figure 6b). All treated groups exhibited a higher speed of migration in the first 8 h compared to the control, although the effect was only significant for the MPE-treated group. At 16 h, the wounds were almost filled with fibroblasts in all samples. Two-way ANOVA showed a significant treatment interaction (*p* = 0.0008 at 8 h) and (*p* = 0.025 at 16 h). Wound closure was very slow after 16 h, without significant differences between the groups. MPE was the most efficient in promoting wound closure. The alginate group showed a lower speed of migration compared to the control. As expected, time had a significant role, as shown by the two-way ANOVA (*p* = 0.0001) analysis in all groups. The nonparametric Kruskal–Wallis test also showed significant differences between the groups (*p* = 0.045). Alginate fibers and hydrogel-based wound dressings are commercially available and are characterized by mechanical strength, antibacterial and anti-inflammatory properties and increased absorption abilities, combined with a low cost of production [43]. However, wounds, especially chronic, nonhealing wounds, are a socio-economic problem that requires long-term medical care and specialized wound healing stimulators. In a previous report, nanoparticles of hydroxyapatite (HAP) and silica (SiO_2_) were combined with alginate fibers to increase their strength and were tested for biocompatibility and wound closure stimulation in keratinocytes and fibroblasts. The data showed an increased wound closure stimulated by the composite fibers [44]. Ag nanoparticles were also added to alginate to increase their antibacterial effect [45]. In our study, the MPE and AlgPE formulations enhanced wound closure by stimulating fibroblast migration, without toxic effects in the cells, which is indicative of a possible stimulatory effect on wound healing *in vivo*.

### 2.6. Oxidative Stress and Inflammation

**Figure 7:**
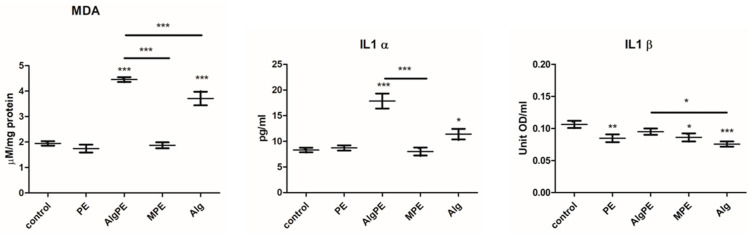
Oxidative stress and inflammation. Malondialdehyde (MDA) was determined spectrophotometrically (TBA method); Il1α and Il1β were measured by ELISA after 48 h of exposure. Data are shown as mean ± SD (*n* = 3). * = *p* < 0.05, ** *p* < 0.001, *** *p* < 0.0001 compared to the control and between the treated groups.

Oxidative damage was assessed by the spectrophotometric measurement of the malondialdehyde, a marker of lipid peroxidation. As seen in Figure 7, the level of MDA was slightly decreased by the PE extract and the MPE formulation, while the AlgPE formulation and alginate strongly increased the lipid peroxidation. There was a significant difference between the MPE and the AlgPE formulations, as well as between the MPE formulation and alginate. There were also significant differences between the groups (*p* = 0.018, Kruskal–Wallis test).

The levels of the inflammatory cytokines IL1α and β were measured by ELISA. The level of IL1 α was strongly increased by exposure to AlgPE and alginate. The level of IL1 β was significantly decreased by PE, MPE and alginate. There were significant differences between the groups (*p* = 0.02 for IL1 α and IL1 β, Kruskal–Wallis test). Based on these findings, the PE–alginate combination had a different effect according to the formulation: AlgPE induced oxidative damage and inflammation by increasing the levels of the proinflammatory cytokine IL1α, while MPE showed a slight anti-inflammatory effect by decreasing the levels of IL1β.

Sodium alginate strongly influenced the biocompatibility of formulations and stimulated the functions of cells [37], particularly fibroblasts. In a previous report, Juniperus leaves and berry extracts were added to an alginate/carboxymethyl cellulose hydrogel to increase their biocompatibility, tissue adhesion and antibacterial activity [46]. Our results showed that sodium alginate-based formulations containing a plantain extract, especially, the MPE formulation, promoted cell viability more effectively than PE, increased the speed of wound closure in a scratch wound assay and reduced oxidative stress and inflammation, leading to a more robust synthesis of extracellular matrix proteins, such as collagen 1 and 3. 

## 3. Conclusions

In our study, the effects of MPE and AlgPE gels on cells were different, despite the gels’ similar chemical composition, depending on the physical structure of the alginate hydrogels. Therefore, the tuning of the alginate structure into spheres and the addition of the plantain extract induced favorable fibroblast responses that stimulated wound closure stimulation.

In conclusion, the developed sodium alginate-encapsulated common-plantain extract is a promising treatment for wound dressing applications.

The future perspectives of this study involve further testing in animal wound healing models of the MPE hydrogel, which represents a basis for the preparation of hydrogels with improved qualities based on synergic mixtures of plant extracts.

## 4. Materials and Methods

### 4.1. Materials

The common plantain used in this study (Plafar SA, Cluj-Napoca, Romania) was purchased from the online market Planteea, a natural and organic products store [47]. Ethanol used for the extractions and methanol were acquired from Chimopar, Bucuresti, Romania and the Folin–Ciocalteu reagent, gallic acid, anhydrous sodium carbonate, 2,20–diphenyl-picrylhydrazyl (DPPH) and 6–hydroxy–2,5,7,8–tetramethyl chroman–2 carboxylic acid (Trolox), used for extract characterization, were bought from Sigma-Aldrich, Heidelberg, Germany. For hydroalcoholic extract encapsulation, we used sodium alginate (Alg) purchased from Sigma-Aldrich, Germany, and calcium chloride dihydrate obtained from VWR Chemicals, Wien, Austria. All reagents used in this study were of analytical grade. The ultrapure water used for the extractions was obtained using a Direct-Q^®^ 3 UV Water Purification System Merck (Darmstadt, Germany).

### 4.2. Preparation and Characterization of the Common-Plantain Extract

The common-plantain extract (PE) was obtained by two methods: infusion and ultrasound extraction. Infusion was conducted using a coffee espresso machine for the electric stove (Mocca, Verk Group, Podolszyn Nowy, Poland) with 10 g of crushed dry plant and 200 mL of ultrapure water. The aqueous PE was left covered until it was cooled to room temperature, after which it was centrifuged for 10 min at 7000 rpm. The extract was stored at 4 °C until further analysis. Also, PE was obtained by sonication of grounded leaves in an Elma ultrasonic bath (Transsonic T 310 at 35 kHz, installed power of 95 W, Singen, Germany) for 30 min at room temperature in ultrapure water. After extraction, the mixture was centrifugated for 10 min at 7000 rpm, and the extract was stored at 4 °C until further analysis.

The obtained extract was characterized in terms of content of total polyphenols by the Folin–Ciocalteu method [48]. To this aim, in a volumetric flask (10 mL) containing 5 mL of ultrapure water, 0.1 mL of extract and 0.5 mL of Folin–Ciocalteu reagent were mixed. The obtained mixture was allowed to stand for 3 min, and then 1.5 mL of Na_2_CO_3_ (5 g L^−1^) was added; a volume of 10 mL was then reached in the volumetric fwith ultrapure water. The flask was kept in a water bath (50 °C) for 16 min, then cooled to room temperature; its absorbance was read relative to a blank sample (ultrapure water). The total polyphenol amount of the extract was calculated using the standard curve of gallic acid equivalent (GAE), drawn in the interval of 0.002–0.8 mg mL^−1^ and expressed in mg GAE/g DW.

The antioxidant capacity of PE was determined according to a slightly modified procedure by Brand-Williams and collaborators [49]. Hence, 0.01 mL of PE was added to 3.9 mL of DPPH radical solution (0.0025 g/100 mL methanol) or to 3.9 mL of methanol (blank sample). The mixtures were left in the dark for 10 min, after which the absorbance at 515 nm was read with a UV–VIS T80 spectrophotometer (PG Instruments Limited, Leicestershire, UK). The results are expressed in mM Trolox/g DW and were calculated from the Trolox calibration curve plotted for different concentrations (0.004–3.2 mM).

### 4.3. Preparation and Characterization of Sodium Alginate Hydrogels

#### 4.3.1. Preparation of Sodium Alginate Hydrogels 

Microencapsulated PE in sodium alginate was prepared by a slightly modified method described by Rijo et al. [50].

(a) A sodium alginate hydrogel (Alg) was prepared by mixing at 600 rpm sodium alginate with ultrapure water in the ratio of 1:32 (*w*/*v*). The stirring took place at 40 °C for 20 min, after which the hydrogel was cooled to room temperature.
(b) Another sodium alginate hydrogel (AlgPE) was obtained in the same way as the first hydrogel, but after it was cooled to room temperature, 5 mL of PE was added under continuous stirring. After adding the extract, stirring continued for another 10 min.
(c) Alginate microspheres (MPE) were obtained by adding the second hydrogel to 200 mL of a 0.05 M CaCl_2_ solution. The hydrogel was added drop by drop with the help of a syringe, under continuous stirring. After finishing adding the hydrogel to the CaCl_2_ solution, stirring was continued for another 15 min. At the end, the formed microcapsules were centrifuged for 5 min at 3000 rpm and washed three times with ultrapure water.

#### 4.3.2. Characterization of the Obtained Microcapsules

For the morphological characterization of the beads, the cold-field emission scanning electron microscope (SEM) Hitachi SU8230 (Tokyo, Japan), operated at 30 kV, was used. The dried/lyophilized beads were covered with a 9 nm layer of platinum prior to the examination.

The recording of the FT-IR spectra of the analyzed samples was carried out with a JASCO 6100 FTIR spectrometer (Tokyo, Japan) in the 4000–400 cm^−1^ spectral domain with 4 cm^−1^ resolution using the KBr pellet technique. Each sample was dispersed in about 300 mg of anhydrous KBr and mixed in an agate mortar. Pellets were obtained by pressing the mixture into an evacuated die. The spectral data were collected and analyzed with Jasco Spectra Manager v.2 software. 

The encapsulation efficiency of PE in sodium alginate was determined according to Pasukamonset et al. [51]. Therefore, the microcapsules (2.5 mg) were sonicated for 30 min with 5 mL of sodium citrate (3% *w*/*v*), after which they were centrifuged for 10 min at 3000 rpm. The total polyphenol content in the supernatant was determined as described in Section 4.2., and the encapsulation efficiency (%) was determined using to the following equation:

(1) $$EE\;(\%)=\frac{{TPC}_{E}-{TPC}_{S}}{{TPC}_{E}}\times 100,$$

where ${TPC}_{E}$ is the total polyphenol content of PE used for encapsulation, and ${TPC}_{S}$ is the total content of non-encapsulated polyphenols.

### 4.4. Biological Assays 

#### 4.4.1. Cell Cultures

The human dermal fibroblasts BJ were purchased from ATCC (BJ-CRL-2522, Gaithersburg, MD, USA) and cultivated in DMEM (Dulbecco’s modified Eagle’s medium) with 5% FBS (Fetal Bovine Serum) and antibiotic and anti-mycotic compounds; all reagents were acquired from Sigma Aldrich, Co. (Heidelberg, Germany).

#### 4.4.2. Alginate Formulations of the Extract 

Samples of the alginate formulations, completely submerged in cell culture medium at a concentration of 2 g mL^−1^, were incubated for 24 h at 37 °C, according to ISO 10993-12/2012 procedures [52]. Then, the obtained extract was sterile-filtered and used immediately for *in vitro* experiments.

#### 4.4.3. Viability Assay

Cell survival was measured by using the CellTiter 96^®^ AQueous Non-Radioactive Cell Proliferation Assay (Promega Corporation, Madison, WI, USA), a colorimetric test that determines the amount of formazan produced by viable cells. Fibroblasts cultivated at a density of 10^4^/wells in 96-well plates (TPP, Trasadingen, Switzerland) were settled for 24 h, then treated with a freshly prepared PE extract with a concentration of 0–100 µg mL^−1^ of polyphenols and with dilutions of the extract in alginate formulations (0.05–1) suspended in medium for 24 h. Viability was measured at 540 nm, using an ELISA plate reader (Tecan, Männedorf, Switzerland). All experiments were performed in triplicate. Fibroblast cultures exposed to the medium were used as controls. Results are presented as % of the viability of the untreated controls; the dose that caused a viability decrease below 70% was considered toxic.

#### 4.4.4. Cell Lysates 

Fibroblasts, seeded in Petri dishes at a density of 10^4^ fibroblasts cm^−2^, were exposed for 48 h to the PM extract containing 5 µg mL^−1^ of polyphenols and to the alginate extract formulations at a dilution of 0.05 (1:20). Untreated cells were used as controls. The longer exposure of 48 h was employed to allow enough time for protein synthesis. Lysates were prepared as previously described [53]. Protein concentration was measured using a DC assay kit according to the manufacturer’s specifications (Biorad, Hercules, CA, USA), using bovine gamma globulin as a standard. 

#### 4.4.5. ELISA and Spectrophotometry

Soluble IL1α (interleukin 1α) and IL1β (interleukin 1β) Quantikine ELISA human immunoassay kits (R&D Systems, Inc., Minneapolis, MN, USA) were used. Quantification of malondialdehyde (MDA), a lipid peroxidation marker, was performed using a malondialdehyde (MDA) colorimetric assay kit (TBA Method) (Elab Science, Wuhan, China). The cell supernatants for the analysis of interleukins and the cell lysate for the MDA assay were treated according to the manufacturer’s instructions; readings were conducted at 450 nm (correction wavelength set at 540 nm), using an ELISA plate reader (Tecan). Results are shown as pg/mL for IL1α, OD/mL for IL1β and nM/mg protein for MDA.

#### 4.4.6. Western Blot

Cell lysates (20 µg protein/lane) were separated by electrophoresis on SDS PAGE gels and transferred to polyvinylidene difluoride membranes, using a Biorad Miniprotean system (BioRad). Primary antibodies against collagen 1 and collagen 3 and the corresponding secondary peroxidase-linked antibodies were bought from Santa Cruz Biotechnology (Dallas, TX, USA). 

Proteins were detected using the Supersignal West Femto Chemiluminiscent substrate (Thermo Fisher Scientific, Rockford, IL, USA) and a Gel Doc Imaging system equipped with an XRS camera and Quantity One analysis software (Biorad, Version 4.6). Glyceraldehyde 3-phosphate dehydrogenase (GAPDH, Santa Cruz Biotechnology) was used as a protein loading control [54].

#### 4.4.7. Scratch Wound Assay 

Fibroblasts were cultivated on 24-well plates at a density of 5 × 10^4^ fibroblasts cm^−2^ and settled for 24 h in medium containing 2% FCS. Then, a linear wound was created by gentle, continuous suction using a sterile pipette tip. Afterwards, the cells were thoroughly washed [55,56] and incubated with medium containing PE at a 5 µg mL^−1^ polyphenol concentration and the alginate extracts (AlgPE, MPE or vehicle Alg) at a dilution of 0.05 (1:20) for 24 h. Each experiment was performed in five replicates. Pictures were taken through an inverted microscope initially and at 8 h, 16 h and 24 h after wound creation, using a 4× objective (bar = 10 µm). The wound area at each time was measured on the photographs using the ImageJ program, MiToBo plugging; each measurement is the average of at least 3 separate measurements. Data are reported as % of wound closure from the initial wound area. 

Calculation: 

% of wound closure at each time point = (initial wound surface − wound surface at the reported time)/initial wound surface *100.

### 4.5. Statistical Analysis

Statistical significance of the differences between control and treated groups was assessed by paired Student’s t-test and two-way ANOVA, followed by Bonferroni posttest and nonparametric Kruskall–Wallis test, using GraphPad Prism version 5.00 for Windows in the case of the scratch wound assay and one-way ANOVA and Kruskal–Wallis test for the inflammatory cytokine, oxidative stress and Western blot assessments (1992–2007 GraphPad Software Inc., San Diego, CA, USA, Version 5 (Trial) 12 March 2007); *p* < 0.05 was considered statistically significant. All reported data are expressed as mean (*n* = 3) ± standard deviation (SD).

## Data Availability

Data are contained within the article.
